# [Corrigendum] Evaluation of the inhibitory effects of vaginal microorganisms on sperm motility *in vitro*

**DOI:** 10.3892/etm.2025.12996

**Published:** 2025-10-16

**Authors:** Huan Wang, Tingtao Chen, Yidan Chen, Tao Luo, Buzhen Tan, Houyang Chen, Hongbo Xin

Exp Ther Med 19:535–544, 2020; DOI: 10.3892/etm.2019.8237

Following the publication of the above article, the authors contacted the Editor to explain that the data panels j and l of Fig. 5A on p. 541, showing the Gram staining results of the adhesion of selected bacteria to sperm cells, contained an overlapping section, such that data which were intended to show the results from differently performed experiments had been derived from the same original source. This error arose as a consequence of the high morphological similarity comparing among different regions, and the mislabeling of certain files during archiving. However, the authors were able to re-examine their original data, and the revised and corrected version of Fig. 5, now showing the correct data for panel 1, is shown on the next page. Note that the error made in assembling this figure did not have an impact on either the results or the conclusions reported in the paper. All the authors agree with the publication of this corrigendum, and are grateful to the Editor of *Experimental and Therapeutic Medicine* for allowing them the opportunity to publish this; moreover, they apologize to the readership for any inconvenience caused.

## Figures and Tables

**Figure 5:**
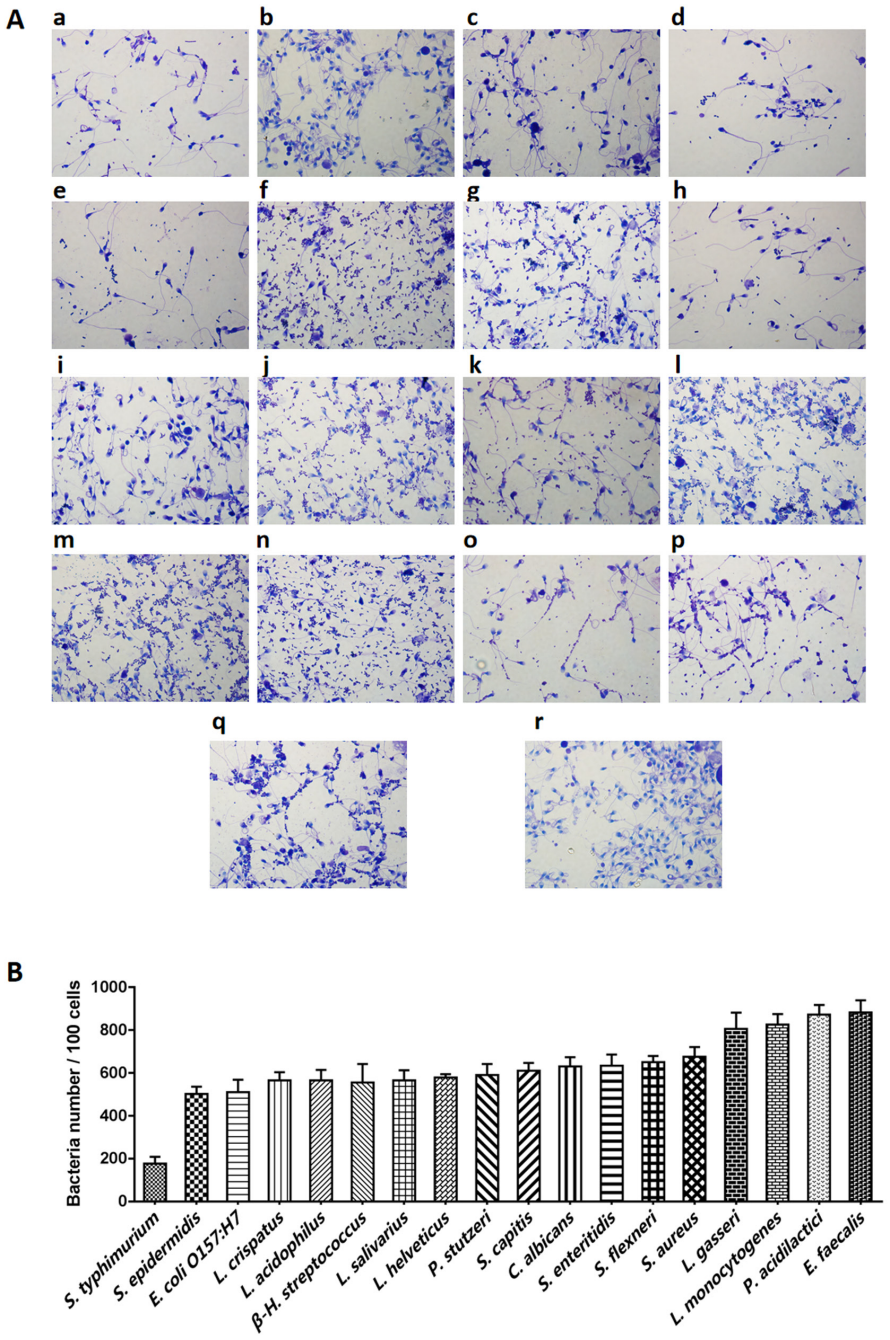
Adhesion of the selected bacteria to sperm cells. (A) The gram stain results (magnification, x100) and (B) adhesion numbers of *S. typhimurium*, *S. epidermidis*, *E. coli O157:H7*, *L. crispatus*, *L. acidophilus*, *β-H. streptococcus*, *L. salivarius*, *L. helveticus*, *P. stutzeri*, *S. capitis*, *C. Albicans*, *S. enteritidis*, *S. flexneri*, *S. aureus*, *L. gasseri*, *L. monocytogenes*, *P. acidilactici and E. faecalis*. The initial sperm/bacteria ratio was 1:10 and *S. epidermidis*, *L. crispatus*, *L. acidophilus*, *L. salivarius*, *L. helveticus*, *P. stutzeri*, *S. capitis*, *L. gasseri* and *E. faecalis* were isolated from vaginal secretions of healthy females, while the others were available inhouse. Statistical analysis was not performed due to the lack of an obvious control strain. (B) Adherence index is defined as the number of adherent bacteria per 100 sperm and was determined from 18 random microscopic fields. Each adherence assay was performed in triplicate. All of the tested bacteria, whether probiotic or pathogenic, adhered to sperms in large numbers.

